# Genomic assisted selection for enhancing line breeding: merging genomic and phenotypic selection in winter wheat breeding programs with preliminary yield trials

**DOI:** 10.1007/s00122-016-2818-8

**Published:** 2016-11-08

**Authors:** Sebastian Michel, Christian Ametz, Huseyin Gungor, Batuhan Akgöl, Doru Epure, Heinrich Grausgruber, Franziska Löschenberger, Hermann Buerstmayr

**Affiliations:** 1 Department for Agrobiotechnology (IFA-Tulln), Institute for Biotechnology in Plant Production, University of Natural Resources and Life Sciences, Vienna (BOKU), Konrad-Lorenz-Str. 20, 3430 Tulln, Austria; 2 Saatzucht Donau GesmbH and CoKG, Saatzuchtstrasse 11, 2301 Probstdorf, Austria; 3 ProGen Seed A.Ş, Büyükdalyan Mah. 2. Küme evler Sok., No: 49, 31001 Antakya, Hatay Turkey; 4 Faculty of Agriculture and Natural Sciences, Department of Field Crops, University of Düzce, 81620 Düzce, Turkey; 5 Probstdorfer Saatzucht Romania SRL, Str. Siriului Nr. 20, Sect. 1, Bucharest, Romania; 6 Plant Breeding Division, Department of Crop Science, University of Natural Resources and Life Sciences, Vienna (BOKU), Konrad-Lorenz-Str. 24, 3430 Tulln, Austria

## Abstract

**Key message:**

**Early generation genomic selection is superior to conventional phenotypic selection in line breeding and can be strongly improved by including additional information from preliminary yield trials.**

**Abstract:**

The selection of lines that enter resource-demanding multi-environment trials is a crucial decision in every line breeding program as a large amount of resources are allocated for thoroughly testing these potential varietal candidates. We compared conventional phenotypic selection with various genomic selection approaches across multiple years as well as the merit of integrating phenotypic information from preliminary yield trials into the genomic selection framework. The prediction accuracy using only phenotypic data was rather low (*r* = 0.21) for grain yield but could be improved by modeling genetic relationships in unreplicated preliminary yield trials (*r* = 0.33). Genomic selection models were nevertheless found to be superior to conventional phenotypic selection for predicting grain yield performance of lines across years (*r* = 0.39). We subsequently simplified the problem of predicting untested lines in untested years to predicting tested lines in untested years by combining breeding values from preliminary yield trials and predictions from genomic selection models by a heritability index. This genomic assisted selection led to a 20% increase in prediction accuracy, which could be further enhanced by an appropriate marker selection for both grain yield (*r* = 0.48) and protein content (*r* = 0.63). The easy to implement and robust genomic assisted selection gave thus a higher prediction accuracy than either conventional phenotypic or genomic selection alone. The proposed method took the complex inheritance of both low and high heritable traits into account and appears capable to support breeders in their selection decisions to develop enhanced varieties more efficiently.

**Electronic supplementary material:**

The online version of this article (doi:10.1007/s00122-016-2818-8) contains supplementary material, which is available to authorized users.

## Introduction

Selection and development of new varieties of autogamous crops relies on a number of different breeding schemes including the pedigree and bulk methods as well as breeding acceleration using doubled haploids or single seed descent with off-season generations. Notwithstanding, they all share a step of conventional phenotypic selection based on preliminary yield trials in their methodology. These preliminary yield trials are for the larger part unreplicated as merely a limited amount of seed is available from each selection candidate at this stage. Although the phenotypic data obtained in this way allow only preliminary predictions of their final values they strongly influence the selection of lines that enter the following more resource-demanding multi-environment trials, a crucial decision in every line breeding program as a large amount of resources are allocated for thoroughly testing these potential varietal candidates.

Genomic selection using genome-wide dense marker maps has been suggested as a more efficient alternative to conventional selection methods (Meuwissen et al. 2001) and several studies have shown its great potential in line breeding to enhance the selection for major agronomic traits like yield both in legumes (Jarquín et al. 2014; Burstin et al. 2015; Tayeh et al. 2015) and small grain cereals (Asoro et al. 2011; Sallam et al. 2015; Spindel et al. 2015; He et al. 2016; Michel et al. 2016). Additionally, genomic selection could support the accumulation of many small effect alleles to provide higher and more durable quantitative disease resistance (Lorenz et al. 2012; Ornella et al. 2012; Daetwyler et al. 2014; Arruda et al. 2015; Rutkoski et al. 2015b), which could be subsequently combined with labor-intensive and costly to assess quality traits (Heffner et al. 2011b; Schmidt et al. 2015).

The broad range of possible applications has led to different strategies concerning the implementation of genomic selection into line breeding schemes (Heffner et al. 2010; Longin et al. 2015; Spindel et al. 2015; Marulanda et al. 2016), though it is generally suggested that a genomic selection step is integrated before multi-environment trials are being conducted. Breeders might thus consider the replacement of traditional preliminary yield trials by genomic selection to spare phenotyping costs or even integrating them into the genomic selection framework as they deliver a first insight into the future performance of the putative varietal candidates (Endelman et al. 2014). An additional concern of genomic selection is the choice of lines that shall constitute the training population (Rincent et al. 2012; Isidro et al. 2015; Marulanda et al. 2015) especially if breeders conduct selection, which is not always optimal for genomic selection models (Zhao et al. 2012). Nevertheless, high quality phenotypic data for multiple traits is usually available for many advanced lines that were already tested in multi-environment trials and could possibly be used to build more suitable training populations. Hence, a comparison between conventional phenotypic selection based on preliminary yield trials and genomic selection together with an appropriate training population design is needed to shed more light on this issue for the optimization and enhancement of line breeding schemes. The objectives of this study were thus to investigate (i) the possibilities and merit of a posteriori training population designs, (ii) integrating phenotypic information from preliminary yield trials into the genomic selection framework and (iii) compare conventional phenotypic selection with various genomic selection approaches in line breeding schemes on the example of bread wheat.

## Materials and methods

### Plant material and phenotypic data

We analyzed a population of 861 genotyped lines from a commercial winter wheat (*Triticum aestivum* L.) breeding program that descend from multiple families and were either in the F_4:6_ generation or directly derived by the double haploid method. Different subpopulations containing 64–192 lines were tested orthogonally in multi-environment trials from 2010 to 2015. Phenotypic data of these lines was thus of high quality, as they were thoroughly tested in all trial locations that spanned from Austria over Serbia, Croatia, Hungary, and Romania to the Central Anatolian High Plateau in Turkey. We also analyzed F_4:5_ generation preliminary yield trials where all lines in the population were pretested in one location and year in Austria from 2011 to 2014 before multi-environment trials were conducted.

Unreplicated earlier generation lines were tested along with replicated check varieties in all trials. The replicated check varieties allowed correcting for spatial field trends according to standard procedure in plant breeding. The entire population of genotyped earlier generation lines from 2011 to 2014 comprised 1203 lines, with 731 lines being unique to the preliminary yield trials. The number of genotyped lines in these preliminary yield trials varied accordingly between 151 and 539 lines as this study also included historical data before genomic selection was routinely implemented into the winter wheat breeding program at hand. Phenotypic records included grain yield (dt ha^−1^) and protein content (%), which was determined by near infrared spectroscopy (NIRS) directly at harvest.

### Statistical analysis of phenotypic data

We followed a two stage analysis strategy of the phenotypic data, where each individual yield trial was analyzed separately in the first stage. Various models correcting for row and/or column effects as well as autoregressive variance–covariance structure of the residuals were introduced (Burgueño et al. 2000) and the best model was chosen by Akaike’s information criterion (AIC) to calculate best linear unbiased estimates (BLUE) for each trial. The heritability was estimated by $h^{2} = \sigma_{G}^{2}/( {\sigma_{G}^{2} + \frac{1}{2}{\text{MVD}}})$, where $\sigma_{G}^{2}$ designates the genetic variance and ${\text{MVD}}$ the mean variance of a difference of the BLUEs (Piepho and Möhring 2007) and trials with a heritability larger than 0.3 were forwarded for further analysis.

Across trial analysis of the multi-environment trials were conducted separately for each year using a linear mixed model of the form:

1 $$y_{ij} = \mu + g_{i} + t_{j} + gt_{ij} + e$$

was fitted for all traits, where $y_{ij}$ are the BLUEs from the first stage, $\mu$ is the grand mean, and $g_{i}$ is the effect of the ith line. The effect of the* j*th trial $t_{j}$ was fixed, while the line by trial interaction effect $gt_{ij} \frac{1}{2}$ was random. The residual variance was fixed and the inverse of the squared standard errors of the means derived from the first stage of analysis were used as weights in this stage to take the varying accuracy of phenotypic records into account (Möhring and Piepho 2009). Additionally, best linear unbiased predictions (BLUP) were derived for preliminary yield trials by modeling a random effect for the inbred lines in which the heritability was estimated by $h^{2} = 1 - \left( {{\text{VD}}_{\text{BLUP}} /2\sigma_{G}^{2} } \right)$ with ${\text{VD}}_{\text{BLUP}}$ being the mean variance of a difference of the BLUPs (Cullis et al. 2006). The replicated check varieties were thereby used to estimate row and column effects as well as the error variance. The individual records of the unreplicated lines could in this way be adjusted accordingly, taking spatial trends in the preliminary yield trials into account. All phenotypic analyses were conducted using the statistical package ASReml 3 (VSN International, 2015) for the R programming environment (R development core team 2016).

### Genotypic data

DNA was extracted following the protocol by Saghai-Maroof et al. (1984) using leaf samples that were collected from F_4:5_ or doubled haploid lines by sampling minimum ten plants per line during early summer. All 861 lines tested in multi-environment trials as well as the 731 lines unique to preliminary yield trials were genotyped using the DarT genotyping-by-sequencing (GBS) approach (Diversity Array Technologies 2015). Quality control was applied by filtering out markers with a call rate lower than 90%, a minor allele frequency smaller than 0.05, and more than 10% of missing data. Missing data of the remaining 6.6 K SNP markers was imputed by an MVN-EM algorithm (Poland et al. 2012). The same marker data was again used for training genomic selection models with F_4:6_ lines. The minor change in average heterozygosity was expected to introduce a small error which was nevertheless seen to be acceptable considering the cost-benefit ratio of re-genotyping all lines in the F_4:6_ generation.

### Genomic selection and estimation of breeding values in preliminary yield trials

Marker effects were estimated using a ridge regression best linear unbiased prediction (RR-BLUP):

2 $${\mathbf{y}} = {\mathbf{Xb}} + {\mathbf{Zu}} + {\mathbf{e}}$$

where ${\mathbf{y}}$ is an Nx1 vector of BLUEs obtained in the phenotypic analysis, ${\mathbf{b}}$ is a vector of F fixed effects and ${\mathbf{X}}$ its corresponding NxF design matrix. ${\mathbf{Z}}$ is a NxM matrix, which coded the M markers as either +1 or −1 for homozygous loci and 0 for heterozygous loci. Random marker effects were assumed to follow a normal distribution ${\mathbf{u}} \sim N\left( {0, {\mathbf{I}}\sigma_{u}^{2} } \right)$ with variance $\sigma_{\text{u}}^{2}$ and ${\mathbf{e}} \sim N\left( {0, {\mathbf{I}}\sigma_{e}^{2} } \right)$. The kinship between lines was estimated by the genomic relationship matrix, which was computed according to Endelman and Jannink (2012):

3 $${\mathbf{K}} = {\mathbf{WW}}^{\text{T}} /2\varSigma \left( {p_{k} - 1} \right)p_{k}$$

where ${\mathbf{W}}$ is a centered NxM marker matrix of the *i* lines with $W_{ik} = Z_{ik} - 2p_{k}$ and $p_{k}$ being the allele frequency at the kth locus. The derived variance–covariance matrix was used to fit mixed linear models of the form:

4 $${\mathbf{y}} = {\mathbf{Xb}} + {\mathbf{Zg}} + {\mathbf{e}}$$

where ${\mathbf{y}}$ is an Nx1 vector of BLUEs obtained in the phenotypic analysis, ${\mathbf{g}}$ is an Nx1 vector of genotypic effects with ${\mathbf{g}} \sim N\left( {0, {\mathbf{K}}\sigma_{G}^{2} } \right)$ and the genetic variance $\sigma_{G}^{2}$ as well as its corresponding random effect design matrix ${\mathbf{Z}}$. The shrinkage parameter was given by $\lambda^{2} = \sigma_{e}^{2} /\sigma_{g}^{2}$ where $\sigma_{e}^{2}$ is the variance of the residuals that followed ${\mathbf{e}} \sim N\left( {0, {\mathbf{I}}\sigma_{e}^{2} } \right)$. The mixed linear models were completed by F fixed effects, which were contained in the vector ${\mathbf{b}}$ and its corresponding NxF design matrix ${\mathbf{X}}$. Fixed effects included years in the case of prediction with multiple years and the grand mean for preliminary yield trials.

Breeding values for all the lines tested in preliminary yield trials were estimated by explicitly entering their phenotypic records i.e., BLUES for grain yield and protein content into model (4). In this way, genetic relationship between the lines were exploited to strengthen the predictiveness of preliminary yield trials although most selection candidates were tested unreplicated in just one plot (Endelman et al. 2014). We like to refer to this method as kinship enhanced best linear unbiased prediction of phenotypic breeding values (KBLUP) in this study to differentiate it from the genomic best linear unbiased prediction (GBLUP) model, where selection candidates are predicted purely on their relationship with a training population without any phenotypic records. Models for estimating marker effects by RR-BLUP were implemented using the R package rrBLUP (Endelman 2011), whereas the GBLUP and KBLUP models for predicting future line performance were fitted with the implementation of ASReml 3 (VSN International 2015) for R (R development core team 2016).

### Cross-validation accuracy and training population design

We first investigated the merit of a posteriori designing a training population by picking a specific set from the entire available population of lines. The phenotypic variance of the training population is a major factor correlated with the prediction accuracy (Isidro et al. 2015; Marulanda et al. 2015), thus we aimed to maximize the phenotypic variance by sampling the highest and lowest performing lines from each respective year for entering into the training population.

The impact of this sampling method on the prediction accuracy was tested by 6-fold cross-validation, where the training and selection populations were built by randomly sampling 20–60 lines from each year and every year constituted a fold. GBLUP models were fitted with randomly sampled training populations and the benefit of maximizing the phenotypic variance was studied by equally sampling lines from the tails of the distribution e.g., the 30 highest and 30 lowest performing lines from a given year. The selection population was always equivalent in both cases and the training population size varied accordingly between 100 and 300 lines. This entire approach corresponds essentially to sampling both genotypes and environments for estimating a less upward biased prediction accuracy of genomic selection than obtained by sampling genotypes alone (Albrecht et al. 2014; Michel et al. 2016). Furthermore, the prediction accuracy of the full data set was estimated by leaving all lines from one year out as validation population and training a GBLUP model with all lines from the remaining 5 years at a time, which resulted in training population sizes of approximately 700 lines and validation populations that were on average composed of 140 lines. The benefit of a posteriori training population design was assessed by sampling 20–90% of the lines from each year in the training population, either randomly or with half of the lines coming again from either tail of the distribution.

### Comparison between conventional phenotypic and genomic selection

The accuracy of conventional phenotypic selection was estimated by correlating the line performance in preliminary yield trials in 2011–2014 and BLUEs from multi-environment trials the following year. This estimate was based on 96–145 retested lines that formed the selection populations and despite a certain selection pressure still covered a broad range of both protein content and grain yield (Fig S1). Line performance per se was thereby predicted by classical BLUP as well as the above described KBLUP that took genetic relationships among lines within preliminary yield trials into account.

Pure genomic selection is on the other hand undertaken without prior knowledge of line performance from preliminary yield trials. We compared this approach with conventional phenotypic selection by predicting the performance of the same 96–145 retested lines but excluded all their phenotypic data from both the year of the preliminary yield trial and the multi-environment trials to fit GBLUP models. The influence of the training population constitution was studied by setting up a cross-validation scheme, using alternatively all possible three-way combinations of the remaining four years in which lines from the selection population did not occur (Fig S2). Hence, every one of the four selection populations was predicted by four different training populations. The training population size was fixed at 180 lines and constructed by sampling an equal number of 60 lines from each one of the training population years. The prediction accuracy of the different selection populations was finally obtained by correlating the genomic estimated breeding values (GEBV) with the BLUEs from the across trial analysis of the multi-environment trials.

### Genomic assisted selection and marker selection

Although genomic selection is a relatively new approach the implementation of preliminary yield trials has been part of most line breeding schemes for a long time. We like to simplify the problem of predicting untested lines in untested years to predict tested lines in untested years in this study by integrating phenotypic information from preliminary yield trials into the genomic selection framework. Therefore, we first estimated the line breeding values by the KBLUP model for every preliminary yield trial and GEBVs from the GBLUP model for every one of the previously described training by selection population combinations. The heritability for the GBLUP model was estimated via the shrinkage parameter $\lambda^{2} = \sigma_{e}^{2} /\sigma_{g}^{2}$ which could be written as:

5 $$\sigma_{e}^{2} /\sigma_{g}^{2} = \left( {1/h^{2} } \right) - 1$$

This approximation by Hofheinz et al. (2012) also allowed us to estimate the heritability *h* ^2^ for the unreplicated preliminary yield trials via both the genetic variance $\sigma_{g}^{2}$ and the residual variance $\sigma_{e}^{2}$ as computed by the KBLUP model. The estimated heritabilities were subsequently used as weights in a heritability index, which was built with predictions from both the GBLUP and KBLUP models:

6 $${\text{GEBV}}_{\text{Index}} = {\text{GBLUP}}_{\text{Scaled}} * {\text{w}}_{\text{GBLUP}} + {\text{KBLUP}}_{\text{Scaled}} * {\text{w}}_{\text{KBLUP}}$$

where ${\text{GEBV}}_{\text{Index}}$ are the GEBVs obtained for genomic assisted selection, ${\text{GBLUP}}_{\text{Scaled}}$ and ${\text{KBLUP}}_{\text{Scaled}}$ are the scaled predictions from the GBLUP and KBLUP models, and the weights ${\text{w}}_{\text{GBLUP}}$ and ${\text{w}}_{\text{KBLUP}}$ are equivalent to the heritabilities computed by (5). The scaling of the prediction was done as appropriate for index selection by subtracting the mean of the predictions and subsequent division by the variance for each GEBV. It should be note that only the selection candidates were involved in the scaling process.

Prior knowledge of line performance from preliminary yield trials enabled furthermore a knowledge-based and more sophisticated selection of markers actually associated with the trait of interest. For this purpose, marker effects were first estimated by fitting RR-BLUP models separately for the preliminary yield trial and the training population of lines in each fold i.e., training by validation population combination of the employed cross-validation scheme. Markers whose effect showed a change of sign between these two models were considered to rather introduce errors into the prediction model and were removed from the marker and genomic relationship matrix before GEBVs were estimated by GBLUP. All phenotypic data involved in the validation of the models was explicitly excluded from this process. RR-BLUP models were also refitted with the selected markers to investigate the proportional change of markers with the same and different sign. We like to highlight at this point that this marker selection approach was only undertaken on the side of the training population from multi-environment trials as no beneficial effect of marker selection was observed when estimating breeding values in preliminary yield trials by KBLUP (data not shown). Assuming larger information content of the GBLUP model in this case the index weight was accordingly adjusted:

7 $$w_{\text{GBLUP}} = h_{\text{GBLUP}}^{2} /\left( {1 - \left| {r_{{{\text{GBLUP}};{\text{KBLUP}}}} } \right|} \right)$$

where $w_{\text{GBLUP}}$ is the index weight, $h_{\text{GBLUP}}^{2}$ the heritability estimated from the GBLUP model following (5) and $\left| {r_{{{\text{GBLUP}};{\text{KBLUP}}}} } \right|$ the absolute value of the correlation between predicted breeding values of lines in selection population based on multi-environment (GBLUP) and preliminary yield trial (KBLUP) data. The adjustment was undertaken as after the marker selection the heritability estimated in the GBLUP model by (5) was reduced, yet a dynamic index with a larger weight on the GBLUP that is based on phenotypic data obtained from several years and locations was seen to be beneficial.

### Selection decision inferences and a one-year selection experiment

After this comparison between selection methods in terms of prediction accuracy we continued by studying their influence on actual selection decisions. An appropriate selection decision by either conventional phenotypic, genomic or genomic assisted selection could be made if lines from preliminary yield trials that are predicted to be among the highest performing lines would also show a superior performance in multi-environment trials. We recorded thus the 5–50% of lines from each training population combination (Fig S2) that were predicted to be among the highest and lowest performing ones by the different selection methods. A comparison was then made whether the conventional phenotypic, genomic or genomic assisted selection approach correctly identified the actual highest and lowest performing lines with a higher frequency averaged over all training by selection population combinations.

Finally, a selection experiment was conducted to test the efficiency of genomic selection compared to conventional phenotypic selection. A set of 60 lines was purely genomically selected in 2013, while the involved wheat breeder selected 70 lines using all available phenotypic information from preliminary yield trials and beyond without genomic information. Among the 60 genomically selected lines 10 lines were chosen for their excellent predicted grain yield, whereas the other 50 were advanced due to superior predicted performance based on a genomic selection index that took grain yield, protein yield as well as fusarium head blight and stripe rust resistance into account (Ametz 2015). The tested set was completed by the five worst performing lines according to the genomic selection index and 31 randomly sampled lines, which were all retested in the multi-environment trials of 2014.

## Results

### Maximizing the phenotypic variance of the training population

**Fig. 1:**
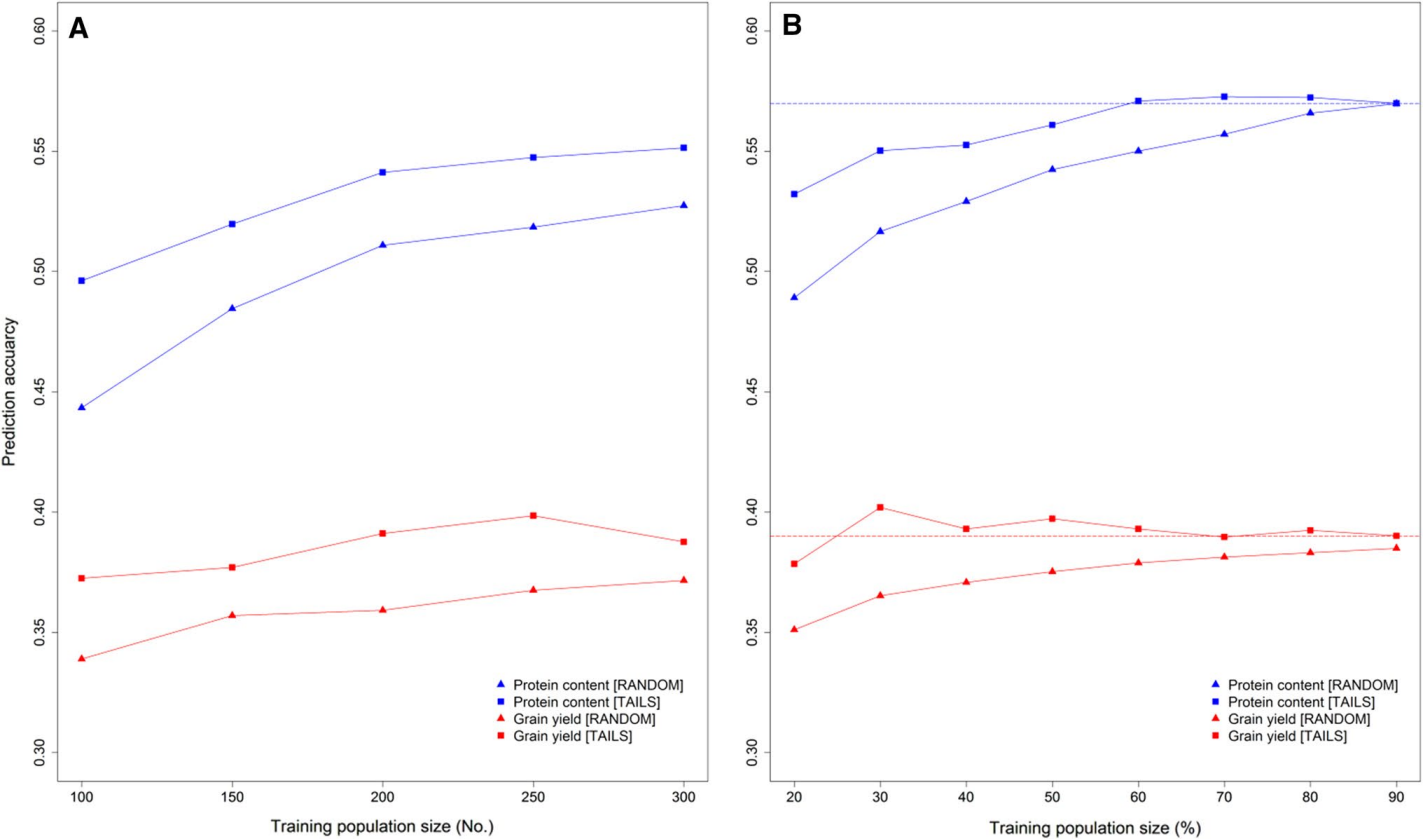
Effect of the training population design on the prediction accuracy for grain yield and protein content. The *lines* in the training population were either randomly sampled or taken from the tails of the distribution, while the selection population was the same set of randomly sampled lines in both designs using a 6-fold cross-validation in which the years constituted the folds (**a**). Leaving all *lines* from 1 year out as validation population sampling 20–90% of the lines from each year in the training population either randomly or with half of the *lines* coming again from the tails of the distribution, where the *dotted horizontal line* designates the average accuracy when training with the entire set of lines of the remaining 5 years (**b**)

We found a classical relationship of higher prediction accuracy with increasing training population size using the 6-years as folds for cross-validation, while this effect was more pronounced for protein content than grain yield (Fig. 1a). The benefit of maximizing the phenotypic variance by sampling the highest and lowest performing lines as training population from each year was minimal in comparison to the full training population when leaving one year out as a validation population at a time (Fig. 1b), while for the 6-fold cross-validation an average increase in prediction accuracy of 7% was observed for both traits. A prediction accuracy of *r* = 0.37 could be reached for example using a randomly sampled training population of 300 lines but was already surpassed when we fitted prediction models with 150 lines from the two tails of the distribution (*r* = 0.38).

The impact of the training population design was also preserved at maximal training population sizes of 300 lines where the accuracy was *r* = 0.55 in comparison to *r* = 0.53 with a random sample for predicting the protein content. Likewise, grain yield was slightly (5%) better predicted using the highest and lowest performing lines for training (*r* = 0.39). The mean accuracies for both sampling methods were furthermore significant different according to a Wilcoxon rank sum test (*p* < 0.01), thus we chose to design training populations consisting of 60 lines from each year with 30 coming from either tail of the distribution to provide a high prediction accuracy with equally sized training populations for all folds in the comparison between conventional phenotypic and genomic selection.

### Predicting the performance of tested and untested lines across years

It is of foremost importance in applied plant breeding programs to select the most promising lines which should enter resource demanding multi-environment trials with a high accuracy to develop successful varieties. We accordingly assessed the correlation between the predicted performance in the year of this selection decision and the actual performance in the following year, utilizing lines that were retested in multi-environment trials 2012–2015.

Classically, lines that will enter more thoroughly testing are selected purely on the basis of phenotypic information from preliminary yield trials. A rather low average prediction accuracy of *r* = 0.21 was found for grain yield using this method, while the highly heritable protein content could be predicted with a reasonable accuracy of *r* = 0.45 (Table 1). The predictive ability of preliminary yield trials could be further enhanced by introducing a genomic relationship to estimate breeding values employing the KBLUP model. Grain yield strongly profited from this method as the accuracy increased by 50% taking the genomic relationships among lines in the unreplicated preliminary yield trials into account.

**Table 1:** Comparison between different selection methods by the prediction accuracy for grain yield and protein content across years, using multi-environment trials (MET), preliminary yield trials (PYT) and the genomic relationship matrix (GRM) as complementing information sources

Selection method	Model	Information source			Prediction accuracy	
		MET	PYT	GRM	Grain yield	Protein content
Phenotypic	BLUP		x		0.21 ± 0.09	0.45 ± 0.08
Phenotypic^†^	KBLUP		x	x	0.33 ± 0.27	0.52 ± 0.14
Genomic	GBLUP	x		x	0.39 ± 0.07	0.50 ± 0.06
Genomic assisted^‡^	GBLUP + KBLUP	x	x	x	0.46 ± 0.07	0.61 ± 0.04
Genomic assisted^§^	GBLUP + KBLUP	x	x	x	0.48 ± 0.05	0.63 ± 0.04

^†^Breeding values based on genetic relationships among lines in unreplicated preliminary yield trials

^‡^Genomic and phenotypic predictions were merged by a heritability index

^§^Markers were pre-selected before fitting the prediction models

Genomic selection on the other hand predicted the performance by the genetic relationship between thoroughly tested lines from multi-environment trials and the younger lines i.e., selection candidates without using any of their phenotypic records. Genomic selection was clearly superior to conventional phenotypic selection and nearly twice the accuracy (*r* = 0.39) could be achieved when predicting grain yield across years with the GBLUP model, whereas approximately the same accuracy was estimated using either GBLUP or KBLUP for protein content.

Both selection methods tackle though different problems: Genomic selection by the GBLUP model is predicting untested lines in untested years with high quality information, while the enhanced phenotypic selection by KBLUP is predicting preliminary tested lines in untested years. Merging the information sources by a heritability index gave a strong advantage over both methods alone, which was 18 and 40% over the GBLUP and KBLUP, respectively, for the low heritable trait grain yield. Even the highly heritable and well predicted protein content benefitted from using this genomic assisted selection approach, resulting in an average prediction accuracy of *r* = 0.61 which was 18–22% better than either the best phenotypic or genomic selection model.

Most astonishing though was the advantage over the conventional phenotypic selection (BLUP). With a prediction accuracy of *r* = 0.46 genomic assisted selection was 119% higher than conventional phenotypic selection for grain yield and gave with *r* = 0.61 also 36% more accurate predictions for the future performance of lines with respect to their protein content. Additionally, this approach gave a higher stability of the prediction accuracy than pure genomic selection by GBLUP as reflected by the lower standard error, and thus narrower confidence interval (Table 1).

**Fig. 2:**
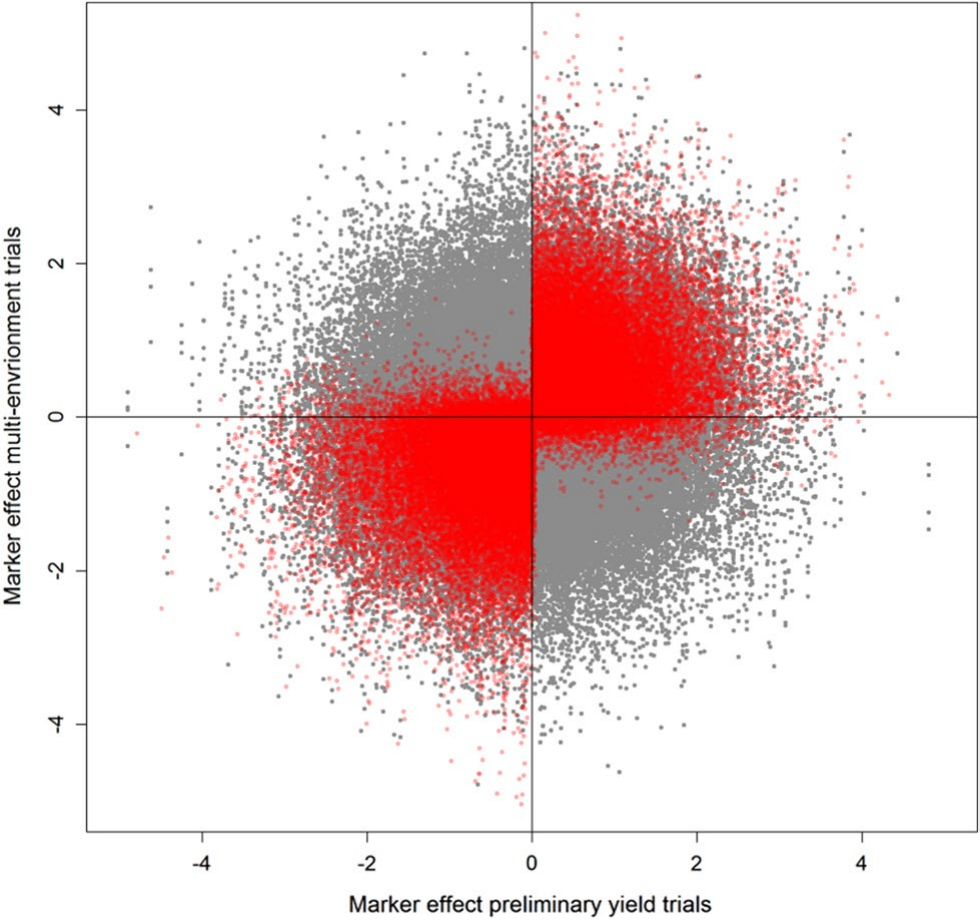
Marker effect estimates before (*grey*) and after (*red*) pre-selection of markers. Marker effects were scaled and centered to allow a comparison between different training by selection population combinations

Prior knowledge from preliminary yield trials gave furthermore the opportunity for a pre-selection of markers associated with the trait of interest in the selection population. Estimation of marker effects by RR-BLUP for both multi-environment and preliminary yield trials separately revealed that around 50% of the marker effects changed their sign between both models, and thus putatively introduced noise when predicting GEBVs (Fig. 2). Removing these markers from the computation of the genomic relationship matrix gave an additional slight increase in prediction accuracy when employing a genomic assisted selection (Table 1).

Interestingly, we found though merely an advantage for pre-selecting markers when it was conducted before fitting GBLUP models but not for the KBLUP which utilized phenotypic records from preliminary yield trials. A noteworthy observation was that after refitting RR-BLUP models with pre-selected markers, some marker effects still showed a change of sign (Fig. 2). Nevertheless, this percentage of putatively noisy markers decreased to 10% resulting in a majority of markers to estimate effects in the same direction.

**Fig. 3:**
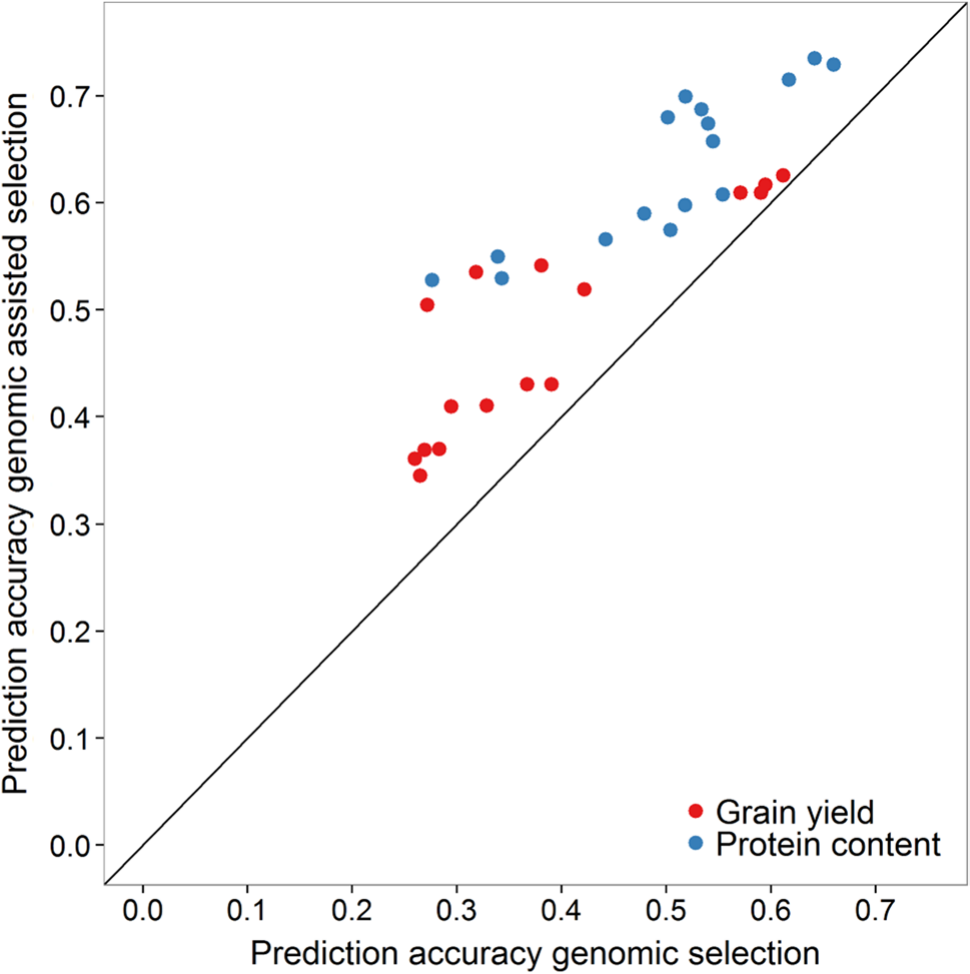
Comparison between the prediction accuracy of genomic and genomic assisted selection for every training by selection population combination to predict grain yield and protein content across years

Genomic assisted selection with additional marker selection also turned out to be a robust approach, which gave constantly higher prediction accuracy than pure genomic selection for all validation by training population combinations (Fig. 3). According to a Wilcoxon rank sum test, the average prediction accuracy of this approach was also significantly higher both for grain yield (*p* < 0.05) and protein content (*p* < 0.01) than what could be achieved by predicting with standard GBLUP alone.

### The influence of genomic assisted selection on selection decisions

The observed high and robust prediction accuracy of the genomic selection approaches promised a reasonably good identification of the highest performing lines in preliminary yield trials for further testing in multi-environment trials. We tested this prospect by examining whether or not the best

**Fig. 4:**
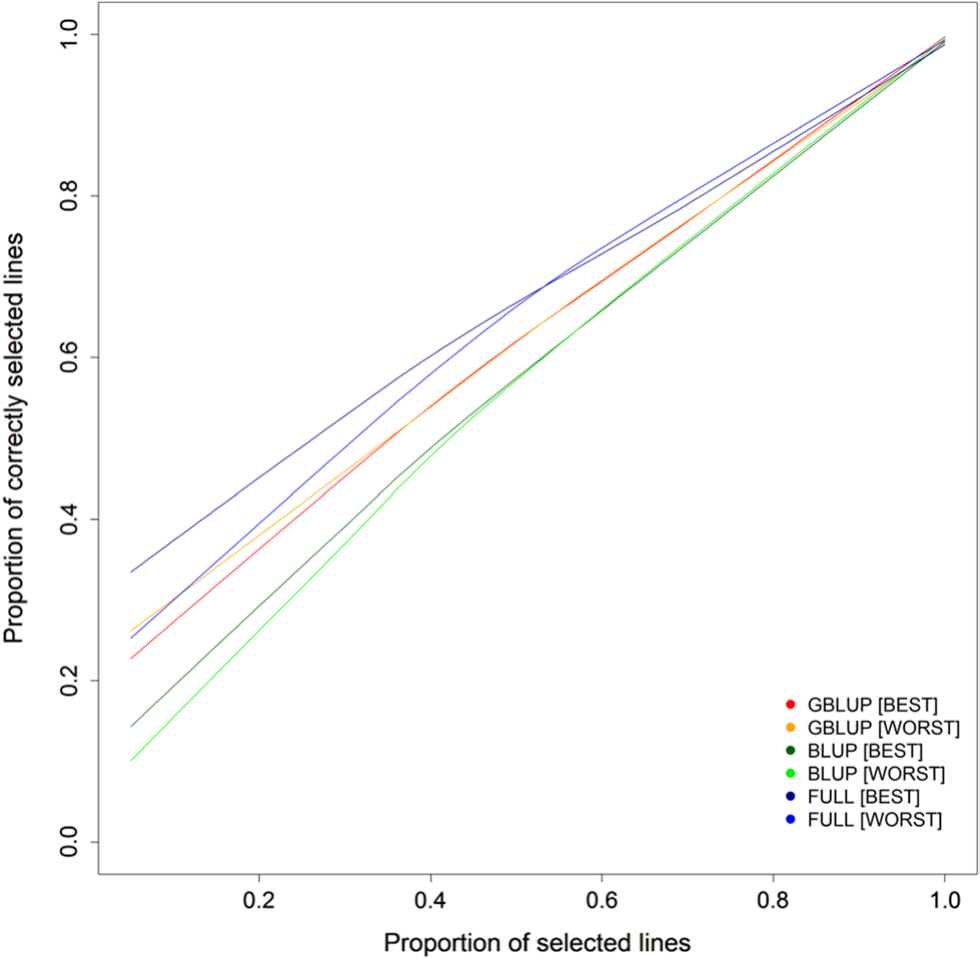
Proportion of correctly selected best and worst performing lines with respect to grain yield by conventional phenotypic selection (BLUP), genomic selection (GBLUP) and genomic assisted selection with pre-selected markers (FULL) at varying selection intensity

10–50% lines according to their prediction were indeed among the best in multi-environment trials. Genomic selection did especially well in this scenario at high selection intensities as applied in typical line breeding schemes and could be improved using a genomic assisted selection with marker selection (Fig. 4).

**Fig. 5:**
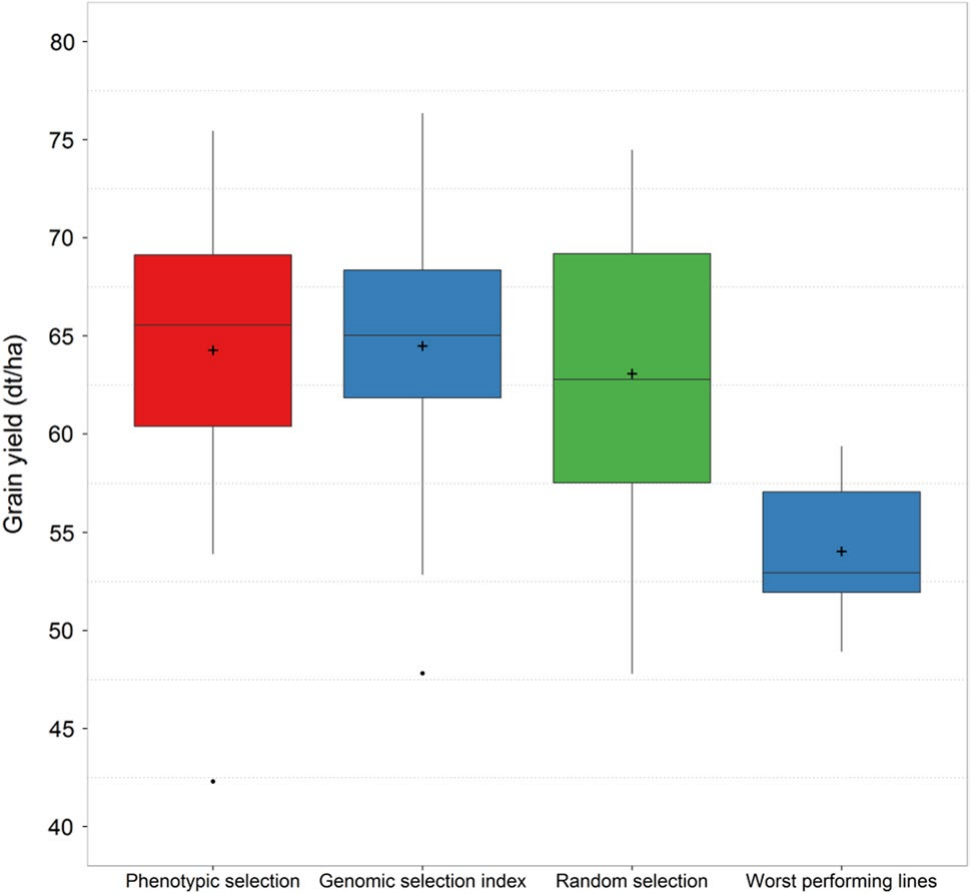
Performance of lines chosen by different selection methods in the selection experiment during the vegetation period 2014

Assuming a breeder would select the best 200 from a total population of 1000 lines (20%), approximately 60 (30%) of these are correctly identified by conventional phenotypic selection but 90 (45%) by genomic assisted selection following the estimates in this study. It is moreover of interest to be informed about the worst lines to discard them by negative selection. This scenario gave nearly orthogonal results to the characterization of the highest performing lines, and the ability to identify the lines from the lower tail of the distribution was verified by the selection experiment (Fig. 5).

Conventional phenotypic selection by the breeder and the genomic selection index performed equally well and surpassed the grain yield of randomly selected lines by 3 dt ha^−1^, which corresponded to a 3% gain by selection. This could be achieved even though the selection index gave a large weight to protein yield i.e., a trait with low prediction accuracy (Michel et al. 2016). Aside from grain yield, the breeder took also a multitude of morphological, quality as well as disease resistance traits into account that are associated with high and stable performance of the selected lines.

## Discussion

This study focused on the prospect of enhancing the efficiency of selection decisions by implementing genomic selection into line breeding schemes. Integrating phenotypic information from preliminary yield trials into the genomic selection framework was combined with a posteriori training population design and resulted in a superior ***genomic assisted selection***. The practical application in commercial bread wheat served as a representative example of this new selection approach.

### A two-tailed training population design

A main driving force of prediction accuracy in genomic selection is the relationship between training and selection population (Clark et al. 2012; Habier et al. 2013; Wientjes et al. 2013). Accordingly, genomic selection is expected to give more accurate predictions if lines included in the training population are closely related to (Asoro et al. 2011; Lehermeier et al. 2014; Lorenz and Smith 2015) or even come from the same population as the selection candidates (Windhausen et al. 2012; Charmet et al. 2014). The underlying population structure can be readily deciphered when multiple large bi-parental populations (Heffner et al. 2011a; Schulz-Streeck et al. 2012; Riedelsheimer et al. 2013; Lehermeier et al. 2014) or larger heterotic groups (Technow et al. 2013; Lehermeier et al. 2014; Spindel et al. 2015) are directly involved in the development of varietal candidates. Training and selection populations in line breeding schemes on the other hand, are usually pre-selected by usage of the pedigree method resulting in small families with varying degree of relatedness. Furthermore, breeders frequently introgress foreign material in their breeding pools and lines are often derived by crosses between introduced and their own germplasm, resulting in an unclear population structure in such mixed line breeding populations (Sallam et al. 2015; He et al. 2016; Michel et al. 2016). Simulation (Habier et al. 2013) and empirical (Lorenz and Smith 2015) studies clearly showed that adding distant relatives to prediction models can have detrimental effects on the accuracy, thus there is serious need for an appropriate training population design to achieve high prediction accuracies with genomic selection in line breeding.

A straightforward approach is the maximization of genetic diversity in the training population on the basis of marker data, which additionally enables to choose a subset of lines before phenotyping and saving costs for field trials (Huang et al. 2013). While this method is applicable to various genomic studies, the choice by the average expected reliability of contrast of lines (CDmean) was especially recommended for genomic selection (Rincent et al. 2012). It was further fine-tuned by Isidro et al. (2015) who integrated breeders’ knowledge about the population structure into their choice of training populations. These approaches as well as the usage of a genetic algorithm based on reliability measures (Akdemir et al. 2015) have shown superior performance for a multitude of traits and crops in comparison to randomly choosing a training population (Rincent et al. 2012; Akdemir et al. 2015; Isidro et al. 2015; Rutkoski et al. 2015a; Tayeh et al. 2015). Marulanda et al. (2015) finally compared more than 21 indices corresponding to eight factors putatively correlated with prediction accuracy in a vast simulation study and found the phenotypic variance to be a major criterion for training population design. Hence, picking individuals from a two-tailed distribution to maximize the phenotypic variance as suggested by Isidro et al. (2015) seems to be a very suitable training population design strategy which was empirically verified in this study.

Notwithstanding, designing training populations a priori based on phenotypic variance might be difficult if the breeding material was not thoroughly tested yet. Moreover, in applied line breeding programs the major goal is to develop new and better performing varieties irrespective of any prediction accuracies. Selecting a posteriori training populations from the numerous potential line varieties in advanced generations might for this reason be a more convenient strategy. Such training populations should preferably include well phenotyped lines that are related to the current selection population and come from both tails of the distribution to ensure a large phenotypic variance. We also recommend to specifically tailoring them for each trait of interest separately, a procedure which is readily realized as the necessary phenotypic data is most cases already available. Even though the beneficial effect of a higher prediction accuracy due to a large phenotypic variance might diminish with increasing training population sizes (Marulanda et al. 2015), models will be computational less burdening but at the same time keeping a high prediction accuracy. Likewise, a two-tailed training population design could guide the choice which lines with historical phenotypic data should be sent to genotyping and might be very useful if few phenotypic records are available for labor-intensive and costly traits such as brewing quality in barley (Schmidt et al. 2015).

Attention should nevertheless be taken if selection is conducted before training populations are built, a common situation in all plant breeding programs that can lead to a strong bias in prediction accuracy of genomic selection approaches (Zhao et al. 2012). The accompanied loss in prediction accuracy could be substantial when carrying out unidirectional selection (Zhao et al. 2012) but usually a broad range of products is developed in line breeding; so even though the population mean is shifted upwards when going into the phase of testing experimental varieties in multi-environment trials a lot of variance from preliminary yield trials is still kept (Fig S1).

### Merging conventional phenotypic and genomic selection

One of the most critical decisions in variety development is the selection of lines that should enter multi-environment trials. The limited phenotypic data that are available for this purpose in early generations led to the suggestion of supporting conventional phenotypic selection by marker assisted selection (Knapp 1998; Lande and Thompson 1990). The implementation of classical marker assisted selection was, however, of limited success for quantitatively inherited traits that are controlled by many loci, while with the advent of genomic selection handling these complex genetic architectures became a much more feasible task in recent years (Jannink et al. 2010; Crossa et al. 2014; Heslot et al. 2015). Although genomic selection has been found to be superior to conventional phenotypic selection and gave outstanding results in several selection experiments (Combs and Bernardo 2013; Beyene et al. 2015; Rutkoski et al. 2015b), genomic predictions rely strongly on genetic relationships and not on physical measurements on the selection candidates.

Hence, preliminary yield trials have the clear advantage of generating solid phenotypic data of which quality can be strongly improved by modeling genetic relationships among the tested lines (Endelman et al. 2014). Integrating pedigree or marker data into the estimation of breeding values has been shown to achieve much higher accuracies when selecting already phenotyped lines in several scenarios (Bauer et al. 2006; Oakey et al. 2007a; Viana et al. 2010; Endelman et al. 2014; Cowling et al. 2015), and was accordingly a very valuable option for enhancing the prediction of line performance across years in this study. The usage of this enhanced phenotypic data from preliminary yield trials for estimating breeding values tackled the problem of predicting tested lines in untested years, while genomic selection usually addresses the more challenging problem of predicting untested lines in untested years.

Merging the before-mentioned merits of genomic selection based on high quality phenotypic data from multi-environment trials with phenotypic selection in preliminary yield trials resulted in a genomic assisted selection that performed much better than either phenotypic or genomic selection alone. The benefits of this approach have also been indicated in bi-parental maize populations for predicting phenotyped doubled haploid lines across years (Lorenz 2013; Riedelsheimer and Melchinger 2013). Krchov et al. (2015) could empirically verify these prospects by combining genomic predictions and phenotypic records with the index weights suggested by Lande and Thompson (1990) for a more accurate prediction of grain yield and moisture in maize hybrids across years. A simple heritability index gave a 12% higher prediction accuracy than the former suggested method in our study, most likely as the additional modeling of a genomic relationship matrix significantly improved the phenotypic data from the preliminary yield trials. The attained genomic assisted selection method resulted furthermore in a higher prediction accuracy for both grain yield and protein content than the other selection approaches, highlighting its superior ability to address the complex inheritance of both low and high heritable traits.

Various marker selection approaches have been proposed for taking the genetic architecture of such traits into account (Heslot et al. 2012; Ogutu et al. 2012; Resende et al. 2012). These efforts are often obstructed by different genetic backgrounds (Schulz-Streeck et al. 2011, 2012) and linkage phase change between the training and selection population (Riedelsheimer et al. 2013; Lorenz and Smith 2015). The incorporation of preliminary yield trials into the genomic selection framework could promote a more targeted pre-selection of marker sets due to prior knowledge of the genetic variation in different selection populations. Hence, we tried to tailor the set of markers fitting the population of selection candidates to account for these altering genetic backgrounds by dropping markers whose effect changed in sign between the training and selection population. Although, we suggest here a rather rough approach that dropped half the markers from the corresponding matrix, the direct pulling of information from preliminary yield trials gave a high and stable average prediction accuracy in combination with genomic assisted selection.

High and stable prediction accuracies are obviously desirable but often very difficult to acquire due to the presence of huge genotype by environment interactions in plant breeding. The prediction of individual trials or locations across years is an especially difficult task (Dawson et al. 2013) and we observed a large variation in prediction accuracy for this undertaking in our study (Fig S3), fitting the results of other studies with autogamous crops (Heslot et al. 2014; Lado et al. 2016). Once multi-environment trials are being conducted, more options open up for enhancing the selection of variety parents like imputing untested lines in tested locations (Burgueño et al. 2011; Jarquín et al. 2014; Crossa et al. 2016; Lopez-Cruz et al. 2015) or enhancing the reliability of breeding values by a relationship matrix (Bauer et al. 2006; Oakey et al. 2007b; Bauer et al. 2009; Müller et al. 2015). Hence, predicting lines for the entire target population of environments might be a better strategy to select candidates that should enter multi-environment trials. These multi-environment trials could afterwards guide selection decisions in breeding for local adaptation to specific regions and variety registration.

### Genomic assisted selection for more sophisticated breeder´s decisions

The chance of selecting the highest performing lines for multi-environment trials was much higher by genomic selection than conventional phenotypic selection in our study, and could be further increased by implementing genomic assisted selection. Depending on the breeding scheme it has been suggested to conduct positive genomic selection for the best lines (Bassi et al. 2016) or discarding the worst lines by negative selection (Longin et al. 2015), while we observed no difference of any genomic selection approach to correctly identify lines from either tail of the distribution. Nevertheless, these considerations are valid for single traits only and it is generally not recommended to sequentially select for one trait after another as a lower gain in selection is expected by such tandem selection (Hazel and Lush 1942). Different multivariate models have been developed to take this problem of simultaneous selection for several traits at the same time into account (Bauer and Léon 2008; Viana et al. 2010; Jia and Jannink 2012).

A computational less demanding alternative could be the usage of genomic selection indices (Ceron-Rojas et al. 2015; Schulthess et al. 2015), and even a simple index based on grain yield, protein content and disease resistance gave a similar gain as conventional phenotypic selection by the breeder in our selection experiment. Genomic selection approaches are thus enabling more sophisticated selection decisions but the knowledge and experience of breeders is still the best guarantee for success, while genomic selection indices can be an additional tool to ease their decisions given the multitude of traits to consider.

## Conclusions

This study showed the strong advantage of genomic selection over conventional phenotypic selection in line breeding schemes on the example of bread wheat. The advantage was further enhanced by a posteriori selecting a training population that maximized the phenotypic variance and the integration phenotypic information from preliminary yield trials into the genomic selection framework. Conducting preliminary yield trials is a common procedure in most line breeding programs, thus we suggested exploiting their information by merging phenotypic and genomic selection for ***genomic assisted selection***. The easy to implement and robust genomic assisted selection gave a higher prediction accuracy than either one of the other methods alone and allowed a more sophisticated selection decision with regard to lines entering multi-environment trials. The proposed method took the complex inheritance of both low and high heritable traits into account and could support breeders in developing varieties that preferably combine high yield, quality, disease resistance and tolerance against abiotic stresses.

### Author contribution statement

SM wrote the manuscript, SM and CA analyzed the data. HGR supported in the statistical analysis. FL, DE, BA and HGU designed the field trials and collected the phenotypic data. FL and HB initiated and guided through the study. All authors read and approved the final manuscript.

## Electronic supplementary material

Below is the link to the electronic supplementary material.

Fig. S1 Variation of grain yield and protein content in preliminary yield trials 2010–2014. (PDF 135 kb)

Fig. S2 Cross-validation scheme used for comparing the different selection methods. Genomic selection models were fitted with training populations of 180 lines, where 60 lines of this training population came from 3 different years (green). Phenotypic and genomic assisted selection included additional data from the year of a preliminary yield trial (orange). All models were validated with a validation population of lines retested in multi-environment trials following the year of a preliminary yield trial (red). (PDF 64 kb)

Fig. S3 Comparison between the prediction accuracy of genomic and genomic assisted selection for every training by selection population combination to predict grain yield and protein content of individual trials across years. (PDF 67 kb)
