# Supplementary material for: Genomic assisted selection for enhancing line breeding: merging genomic and phenotypic selection in winter wheat breeding programs with preliminary yield trials
Source: Theor Appl Genet. 2016 Nov 8;130(2):363–76. doi: 10.1007/s00122-016-2818-8 (PMC5263211; doi:10.1007/s00122-016-2818-8)
Supplement: Supplementary file 1 — Fig. S1 Variation of grain yield and protein content in preliminary yield trials 2010–2014. (PDF 135 kb) [file 122_2016_2818_MOESM1_ESM.pdf]

**Online Resource 1**

**Article Title:** Genomic Assisted Selection for Enhancing Line Breeding: Merging Genomic and Phenotypic Selection in Winter Wheat Breeding Programs with Preliminary Yield Trials

**Journal**: Theoretical and Applied Genetics

**Authors**: Sebastian Michel, Christian Ametz, Huseyin Gungor, Batuhan Akgöl,Doru Epure, Heinrich Grausgruber, Franziska Löschenberger, Hermann Buerstmayr

**Name, affiliation, and email of corresponding author:**

Hermann Buerstmayr
Department for Agrobiotechnology (IFA-Tulln)
Institute for Biotechnology in Plant Production
University of Natural Resources and Life Sciences, Vienna (BOKU)
Konrad-Lorenz-Str. 20, 3430 Tulln, Austria
e-mail: hermann.buerstmayr@boku.ac.at

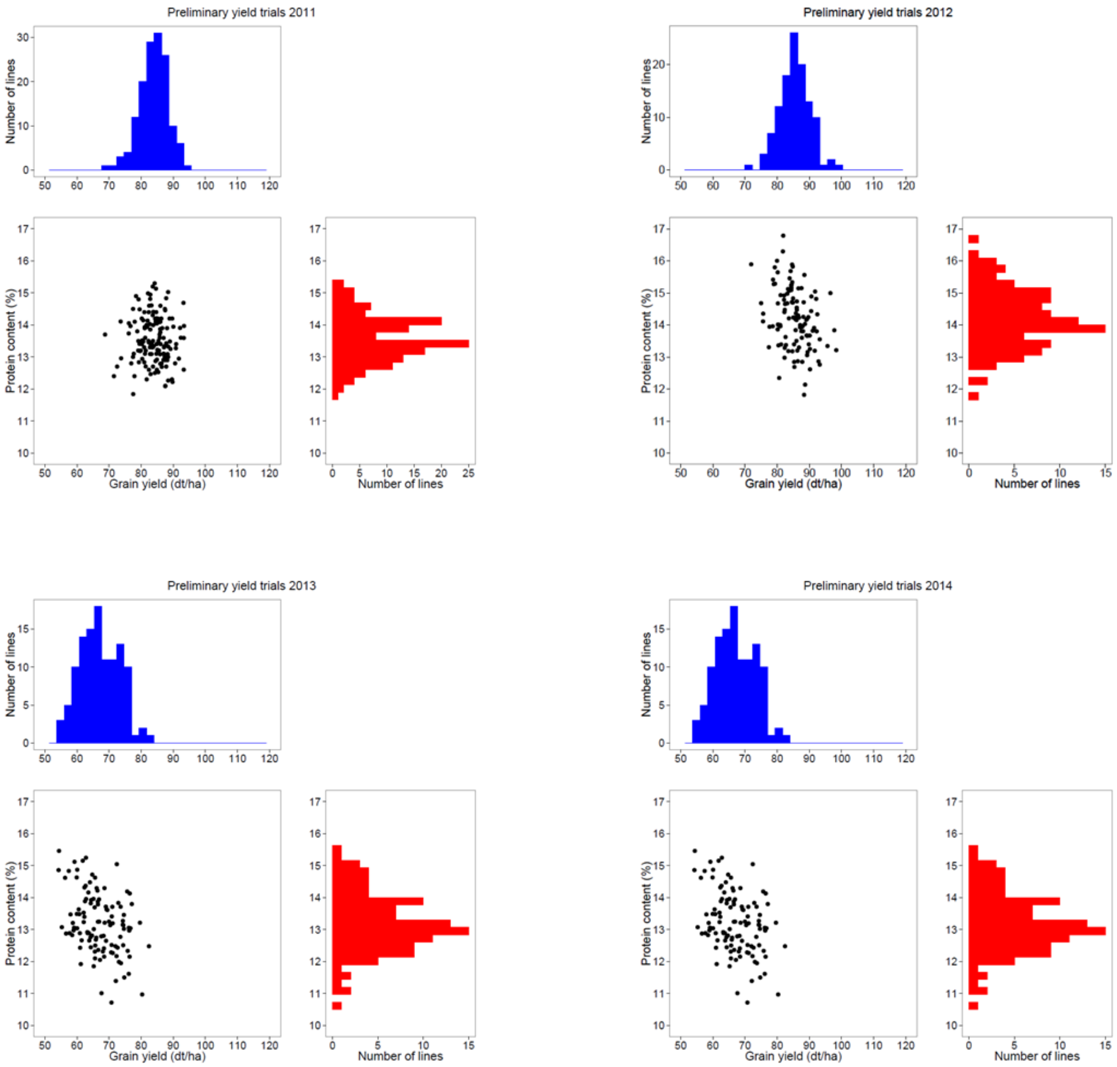


**Fig. S1** Variation of grain yield and protein content in preliminary yield trials 2010-2014.
