# Supplementary material for: Genomic assisted selection for enhancing line breeding: merging genomic and phenotypic selection in winter wheat breeding programs with preliminary yield trials
Source: Theor Appl Genet. 2016 Nov 8;130(2):363–76. doi: 10.1007/s00122-016-2818-8 (PMC5263211; doi:10.1007/s00122-016-2818-8)
Supplement: Supplementary file 2 — Fig. S2 Cross-validation scheme used for comparing the different selection methods. Genomic selection models were fitted with training populations of 180 lines, where 60 lines of this training population came from 3 different years (green). Phenotypic and genomic assisted selection included additional data from the year of a preliminary yield trial (orange). All models were validated with a validation population of lines retested in multi-environment trials following the year of a preliminary yield trial (red). (PDF 64 kb) [file 122_2016_2818_MOESM2_ESM.pdf]

Online Resource 2

Article Title: Genomic Assisted Selection for Enhancing Line Breeding: Merging Genomic and Phenotypic Selection in Winter Wheat Breeding Programs with Preliminary Yield Trials

Journal: Theoretical and Applied Genetics

Authors: Sebastian Michel, Christian Ametz, Huseyin Gungor, Batuhan Akgöl,Doru Epure, Heinrich Grausgruber, Franziska Löschenberger, Hermann Buerstmayr

Name, affiliation, and email of corresponding author:

Hermann Buerstmayr
Department for Agrobiotechnology (IFA-Tulln)
Institute for Biotechnology in Plant Production
University of Natural Resources and Life Sciences, Vienna (BOKU)
Konrad-Lorenz-Str. 20, 3430 Tulln, Austria
e-mail: hermann.buerstmayr@boku.ac.at

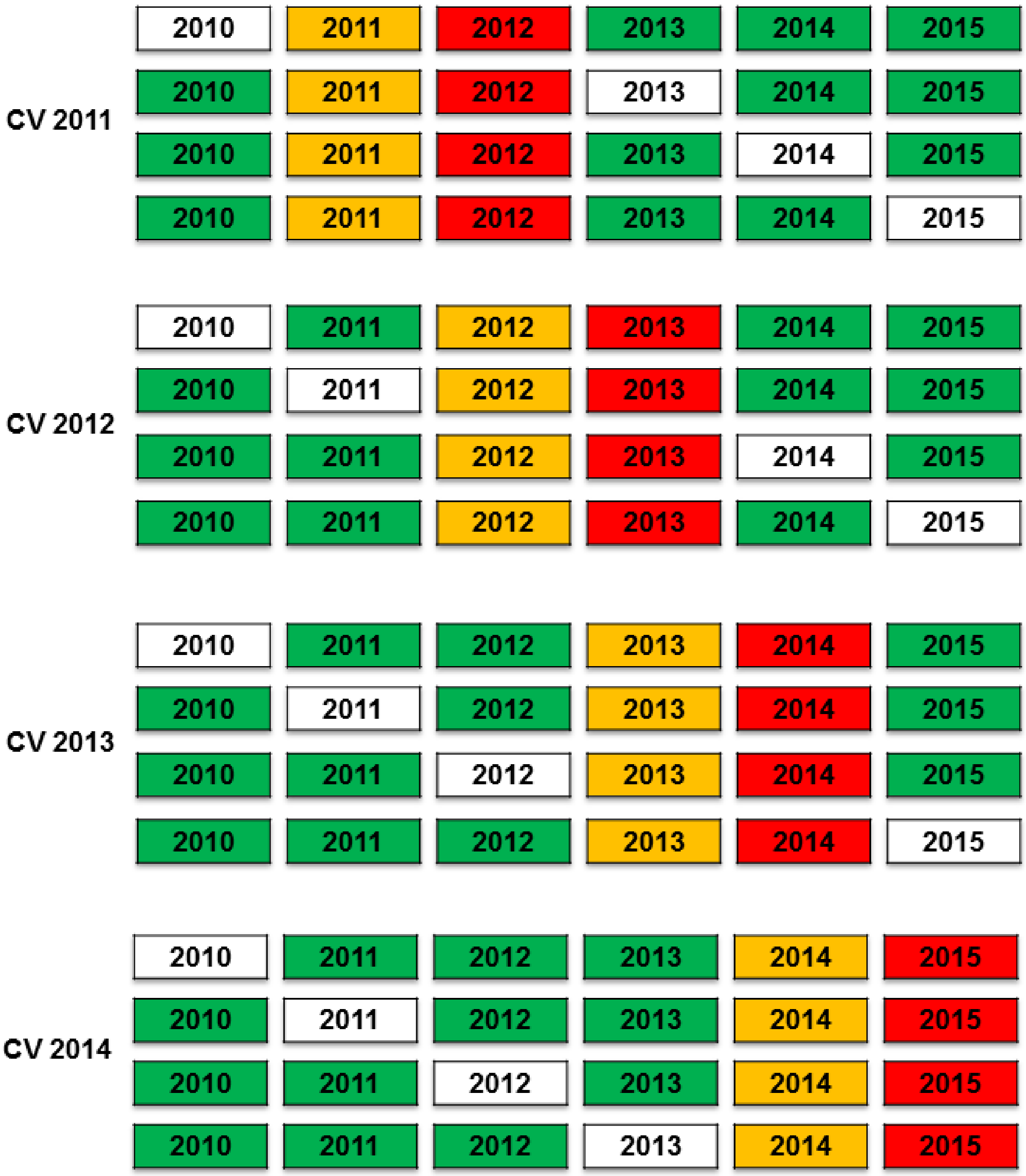

Fig. S2 Cross-validation scheme used for comparing the different selection methods. Genomic selection models were fitted with training populations of 180 lines, where 60 lines of this training population came from 3 different years (green). Phenotypic and genomic assisted selection included additional data from the year of a preliminary yield trial (orange). All models were validated with a validation population of lines retested in multi-environment trials following the year of a preliminary yield trial (red).
