# Supplementary material for: Genomic assisted selection for enhancing line breeding: merging genomic and phenotypic selection in winter wheat breeding programs with preliminary yield trials
Source: Theor Appl Genet. 2016 Nov 8;130(2):363–76. doi: 10.1007/s00122-016-2818-8 (PMC5263211; doi:10.1007/s00122-016-2818-8)
Supplement: Supplementary file 3 — Fig. S3 Comparison between the prediction accuracy of genomic and genomic assisted selection for every training by selection population combination to predict grain yield and protein content of individual trials across years. (PDF 67 kb) [file 122_2016_2818_MOESM3_ESM.pdf]

Online Resource 3

Article Title: Genomic Assisted Selection for Enhancing Line Breeding: Merging Genomic and Phenotypic Selection in Winter Wheat Breeding Programs with Preliminary Yield Trials

Journal: Theoretical and Applied Genetics

Authors: Sebastian Michel, Christian Ametz, Huseyin Gungor, Batuhan Akgöl,Doru Epure, Heinrich Grausgruber, Franziska Löschenberger, Hermann Buerstmayr

Name, affiliation, and email of corresponding author:

Hermann Buerstmayr
Department for Agrobiotechnology (IFA-Tulln)
Institute for Biotechnology in Plant Production
University of Natural Resources and Life Sciences, Vienna (BOKU)
Konrad-Lorenz-Str. 20, 3430 Tulln, Austria
e-mail: hermann.buerstmayr@boku.ac.at

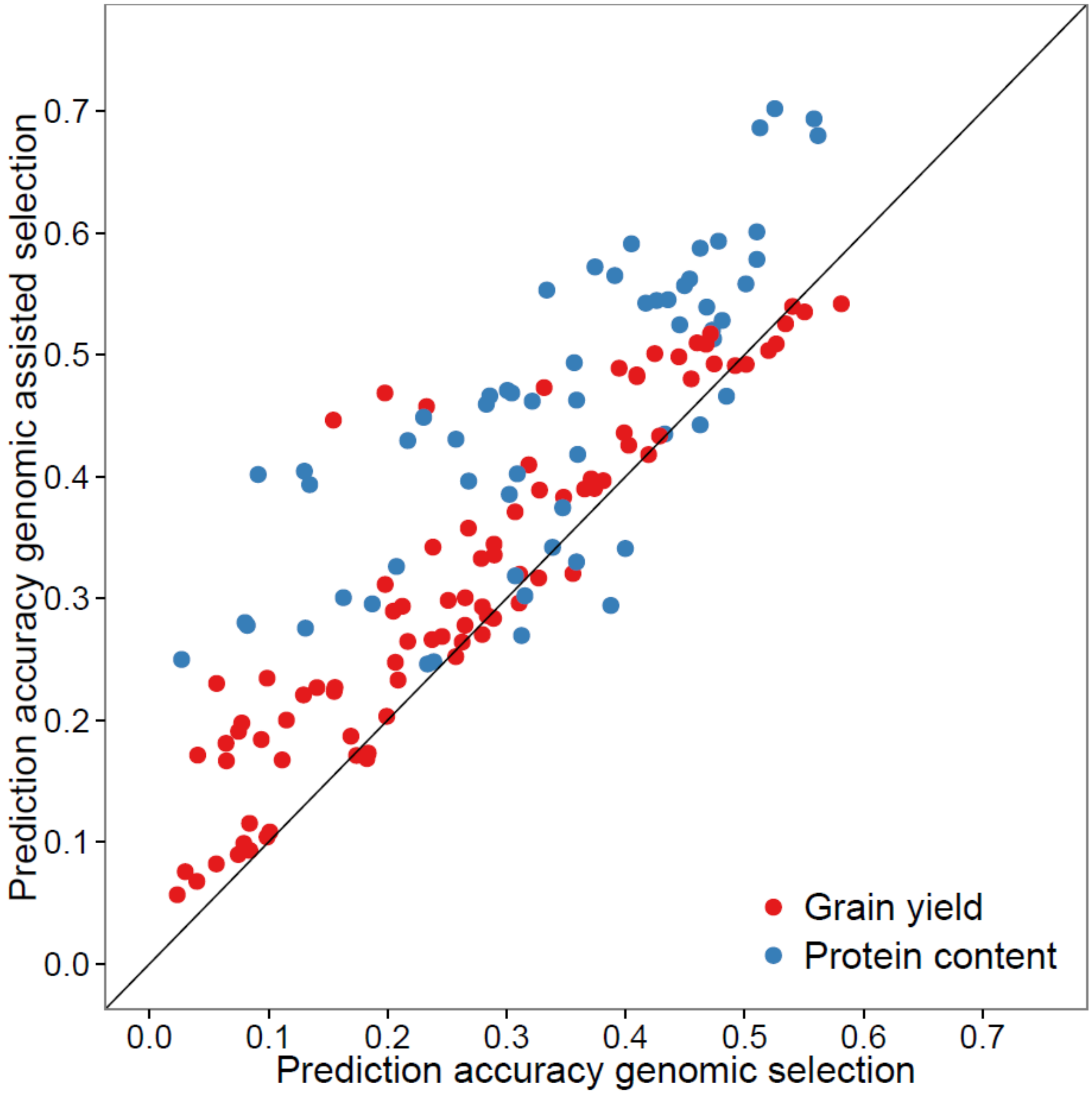

Fig. S3 Comparison between the prediction accuracy of genomic and genomic assisted selection for every training by selection population combination to predict grain yield and protein content of individual trials across years.
